# Indoors Locality Positioning Using Cognitive Distances and Directions

**DOI:** 10.3390/s17122828

**Published:** 2017-12-07

**Authors:** Yankun Wang, Hong Fan, Ruizhi Chen

**Affiliations:** 1 State Key Lab for Information Engineering in Surveying, Mapping and Remote Sensing, Wuhan University, 129 Luoyu Road, Wuhan 430079, China; yankun.wang@whu.edu.cn (Y.W.); ruizhi.chen@whu.edu.cn (R.C.); 2 Collaborative Innovation Center of Geospatial Technology, Wuhan University, Wuhan 430079, China

**Keywords:** spatial relationships, membership function, positioning localities indoors, locality description

## Abstract

Spatial relationships are crucial to spatial knowledge representation, such as positioning localities. However, minimal attention has been devoted to positioning localities indoors with locality description. Distance and direction relations are generally used when positioning localities, namely, translating descriptive localities into spatially explicit ones. We propose a joint probability function to model locality distribution to address the uncertainty of positioning localities. The joint probability function consists of distance and relative direction membership functions. We propose definitions and restrictions for the use of the joint probability function to make the locality distribution highly practical. We also evaluate the performance of our approach through indoor experiments. Test results demonstrate that a positioning accuracy of 3.5 m can be achieved with the semantically derived spatial relationships.

## 1. Introduction

The importance of spatial relationships is well recognized in many domains, such as image interpretation, spatial reasoning, and spatial knowledge representation [1,2,3,4]. Spatial relationships in geographical information systems (GIS) commonly involve three components, namely, a distance relation (e.g., “near” and “100 m”), a direction relation that describes angle order, and a topological relation that describes intersections and neighborhoods [5].

Locality description contains one or more place names and spatial relationships. Any natural or artificial feature with a name can be used as a reference object (RO) [5,6,7]. Locality description can be derived from daily communication; for example, we could state that “object A is 100 m north, and object B is 50 m northwest” for outdoor cases or “object A is 50 m in front, and object B is 30 m to the right” for indoor cases. Humans tend to use relative direction to describe locality. Semantically derived direction and distance are frequently combined to perform a highly precise locality description [5]. The mapping mechanism between qualitative and quantitative data is a research issue not only in GIS but also in other domains [8]. The current work introduces a novel method of positioning localities indoors by locality description.

**Figure 1:**
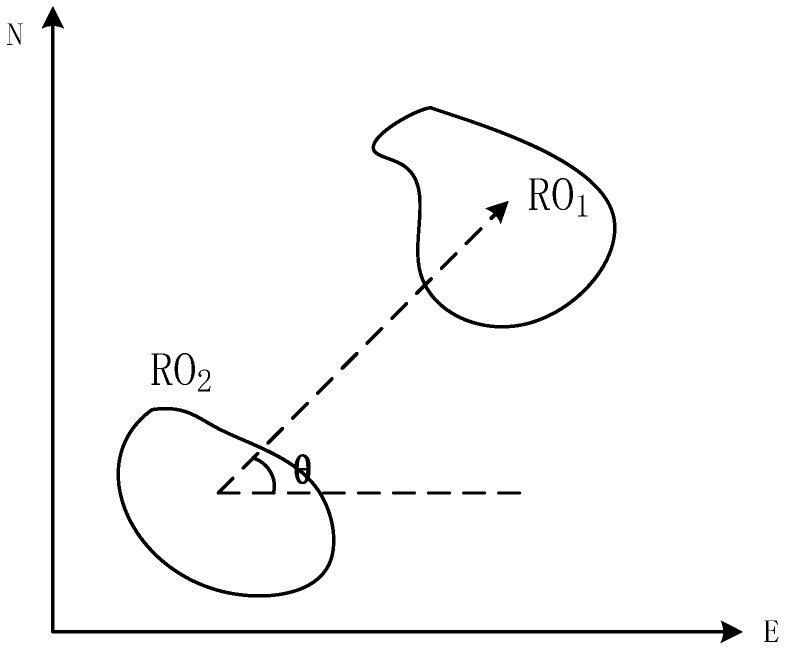
Relative angle of ROs.

Existing literature on this topic includes many meaningful references for positioning localities by locality description. To deal with uncertainties associated with point localities, Wieczorek [9] developed a method to combine different types of uncertainty into a point radius. Guo [10] considered the shape of ROs, refined Wieczorek’s method, and proposed a probabilistic approach for georeferenced localities for museum data collection. Liu [5] extended the work of Guo and developed a general method to position localities through spatial assertion. The author indicated that when distance is quantitative, which may be uncertain, the target object (TO) is distributed in the band surrounding the RO. A probability density function (PDF) was provided for absolute direction relations to describe the probability distribution of a locality. Furthermore, refinement and integration were utilized for cases in which two or more ROs were involved in 2D Euclidean space. This method is suitable for various locality descriptions, but the indoor locality description (i.e., “object A is 50 m in front, and object B is 30 m to the right”) described in this work involves relative direction relations. In other words, direction relations, ROs, and descriptive target locality should be transformed from relative to absolute if the method proposed in [5] is used. Calculating the relative angle of ROs (Figure 1) and descriptive target locality can resolve this problem. Several studies have indicated that a histogram of angles can represent fuzzy relative direction relations between objects [11,12]. However, ROs are crisp objects, and the region of target locality description is a fuzzy object with an uncertain distribution, which is inappropriate. Even if such a relationship is appropriate, the calculation cost would be high. Accordingly, other breakthroughs have been presented. The “between” relations provided in [1,13] indicate that human perception of the spatial relation between two objects is closely related to angular information, which conforms to the trapezoid membership function.

Our novel contribution is that we introduce a method to position localities indoors via cognitive distances and directions. The method, which is based on a joint probability function, consists of distance and relative direction membership functions. Definitions and restrictions are proposed to make the process of positioning localities conform to cognition. The test results demonstrate that a positioning accuracy of 3.5 m can be achieved with cognitive distances and directions.

The paper is organized as follows: in Section 2, we review previous studies on spatial relationships. In Section 3, we conduct a recognition experiment on distance to construct a distance membership function and propose a membership function for relative direction. In Section 4, we develop a method of positioning localities with a joint probability function on the basis of distances and directions. Examples are presented to demonstrate the method of positioning localities in Section 5, and the conclusions and directions for future work are provided in Section 6.

## 2. Related Work

The definitions of spatial relations in existing literature are briefly presented in this section.

### 2.1. Distance Relationship

Distance relationship may be divided into qualitative and quantitative when describing localities [5]. “Near”, “far”, and other statements are used to describe qualitative distance relationships, and qualitative distance provides a rough clue about locality. Worboys [14] conducted a cognitive experiment on the vague spatial relation “near” in environment space and concluded that formal theories can be applied to reasoning with vague spatial notions. In [15], a statistical approach called ordered logit regression was used to predict metric distance on the basis of corresponding context information and linguistic distance.

**Figure 2:**
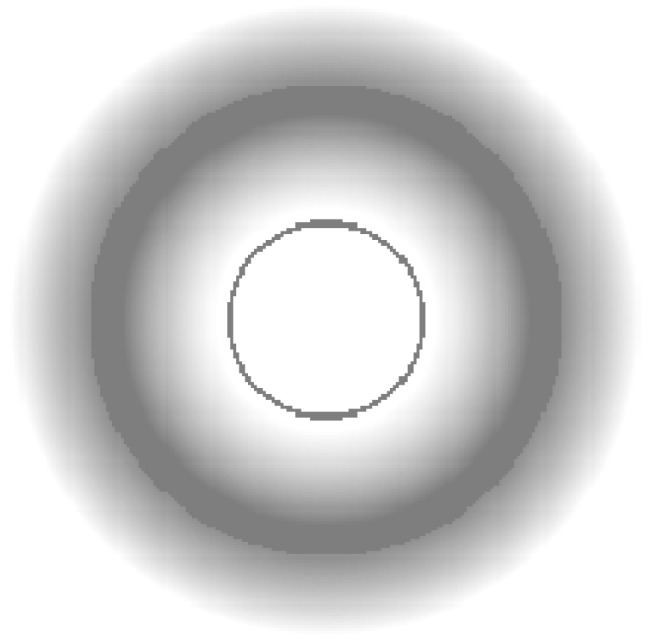
Illustration of uncertain quantitative distance and error following a normal distribution [5].

Compared with qualitative distance, quantitative distance, also called semi-quantitative distance, is utilized more frequently and thus conveys more accurate information on locality [5]. Liu comprehensively discussed quantitative distance and argued that distance may be uncertain because of measurement errors or imprecise records, and different uncertainties possess different probability distributions; among these uncertainties, that caused by a measurement error (Figure 2) has a normal distribution. Considering that our cognition of spatial distance is consistent with that involving a measurement error, we adopt this model and discuss it in Section 3.1.

### 2.2. Direction Relationship

**Figure 3:**
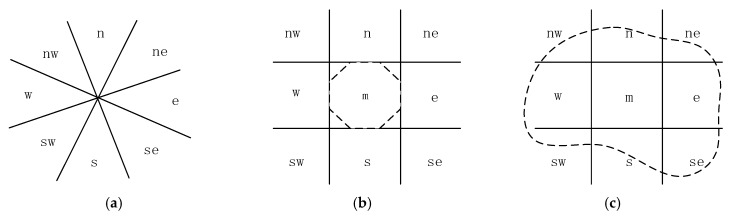
(**a**) Cone-based CDR model; (**b**) MBR-based CDR model; (**c**) MBR-based ICD model (the dashed line is the reference object, and the solid lines are the boundaries of directions).

Direction relationship can be categorized as relative (i.e., front and back) and absolute (i.e., north and south) when describing locality. Cardinal direction relationships (CDR) (Figure 3) are influential relationships abstracted from angle values that divide a space into cones. Describing a locality is complicated because of the vagueness of the direction relationship. Many direction models have been developed; these models include cone based (Figure 3a) [16], which is applicable to point ROs; minimal bounding rectangle (MBR) (Figure 3b) [17], which is suitable for linear or areal ROs; and internal cardinal direction (Figure 3c) [18], which refines the spatial relationship when the TO is inside an areal RO.

Fuzzy mathematical relations are utilized to describe the relative positions of objects. Deng [11] proposed of the use of a histogram of angles to represent the relative positions of objects. However, this approach has a high computational cost and considers only the raster date. Matsakis [12] introduced the histogram of force, which is superior to the histogram of angles and can process vector data. Since then, numerous studies have been conducted based on this notion [2,19,20].

**Figure 4:**
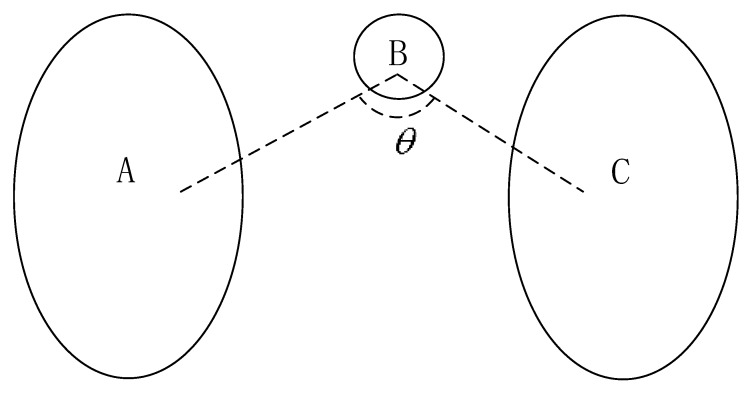
Illustration of the definition “between” in [1].

Several interesting relations, such as “left”, “between”, and “above”, were mentioned in [1]. The authors argued that human perception of spatial positions between two objects is approximately related to angle information and defined relative direction membership functions for them. We use the “between” relationship (Figure 4) as an example. The degree to which object B is between two objects A and C is calculated based on point relations. For all points with a ∈ A, b ∈ B, and c ∈ C, angle Θ at b with edges that connect a and c is calculated. A membership function is proposed to illustrate the degree to which b is between a and c. For extended objects, angle Θ is the average angle over points (a, b, c). By considering the shape of an object, [15] developed the “between” relation and proposed the definition of visibility. Other concepts, such as “along” and “surrounding” [21,22], have been developed from these concepts. All concepts are based on an angle between two sight lines.

## 3. Fuzzy Distance and Relative Direction Function

We conduct a cognitive experiment to construct a distance membership function and construct a fuzzy relative direction model.

### 3.1. Cognitive Experiment and Fuzzy Distance Function

A cognitive experiment on distance is conducted in a shopping market, which is an ideal indoor environment that provides sufficient participants of different ages and backgrounds. During the experiment, the participants are asked to stand at a marked point and describe the distance between the marked point and cognitive objects (shops in the market). Through field work on the shopping market, we established three groups of cognitive distance experiments at 10, 30, and 50 m, with each group containing 40 participants. We established rules for the cognitive experiment in consideration of the factors that influence distance cognition [15]. Each group is expected to locate three cognitive objects (one or two other adjacent objects and isolated) to avoid the influence of spatial distribution of objects. The orientation between the cognitive objects and participants is arbitrary (either positive or inclined) to simulate the real environment. Each participant is sampled once to prevent the cross influence of different groups.

**Figure 5:**
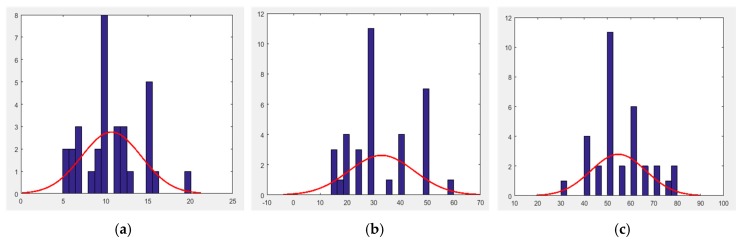
Normal distribution of fuzzy distance cognition. (**a**) 10 m; (**b**) 30 m; (**c**) 50 m.

Data that deviate significantly from the correct distance are eliminated. For example, if the correct distance is 10 m, but the cognitive distance is 30 m, which deviates significantly from the correct distance and does not follow the normal distribution. As shown in Figure 5, cognitive distance follows a normal distribution after normality examination. A large cognitive distance equates to a large deviation from the correct distance.

We will use fuzzy sets to represent the vague geographical knowledge. A fuzzy set in a universe X is formally defined as a mapping U from X to the unit interval [0, 1]. For x in X, U(x) is called the membership degree of x in U, and reflects the extent to which x has the (fuzzy) property that U is modeling. Fuzzy sets are particularly useful to represent fuzzy distance relations.

When used to define fuzzy distance relations, the trapezoid function presents many advantages, such as computation efficiency, robustness, and intuitiveness [23]. Numerous qualitative and semi-qualitative distance relationships can be defined with a trapezoid function [5,11,23].

We let α, β, γ and δ be non-negative numbers, and the order is α ≤ β ≤γ ≤ δ. On the basis of the cognitive experiment, we model fuzzy distance as a non-isosceles trapezoid membership function μ_dis_(d).

(1) $$\mu_{\mathrm{dis}}(d)=\{\begin{array}{c} 1\\ \left(\beta -\alpha )(d-\alpha \right)\\ \left(\gamma -\delta )(d-\delta \right)\\ 0 \end{array}\;\begin{array}{c} \beta \leq d\leq \gamma \\ \alpha \leq d\leq \beta \\ \gamma \leq d\leq \delta \\ d\leq \alpha ,\delta \leq d \end{array}$$

**Figure 6:**
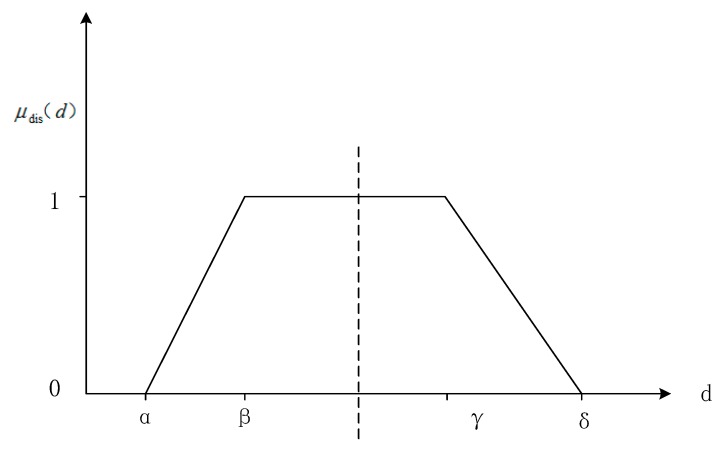
Illustration of the fuzzy distance membership function. In Equation (1), β and γ are the deviation from the correct distance, and α and δ may be derived from the fuzzy distance distribution.

An illustration of the fuzzy distance membership function is shown in Figure 6.

### 3.2. Fuzzy Relative Direction Function

Human perception of the spatial relation between two objects is closely related to angular information [1]. For instance, a person could search a cone area by turning approximately 45° from front to front–left; such a process does not involve distance. On the basis of the relative direction membership function for “right”, “left”, and “above” constructed in [1], we define the membership function for fuzzy relative direction as $\mu_{\mathrm{reldir}}(\Theta )$:

(2) μreldir(Θ)={1π8+|π4×path(Θ)−Θ|π8−a0 |π4×path(Θ)−Θ|≤aa≤|π4×path(Θ)−Θ|≤π8|π4×path(Θ)−Θ|≥π8

**Figure 7:**
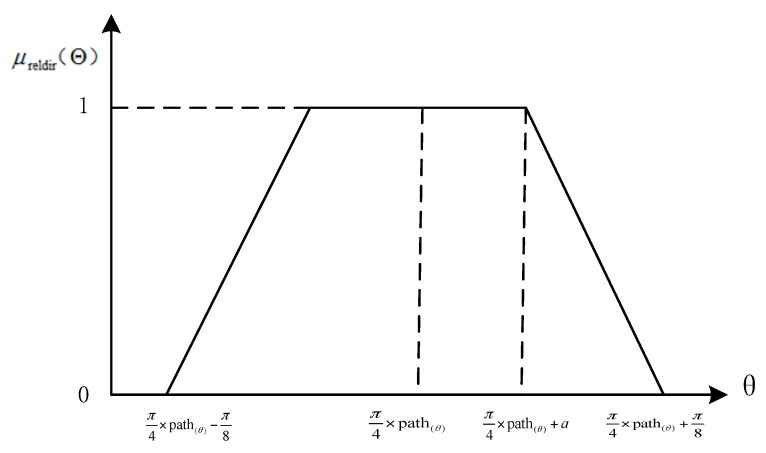
Illustration of the fuzzy relative direction membership function, Equation (2).

An illustration of the fuzzy relative direction membership function is provided in Figure 7.

**Figure 8:**
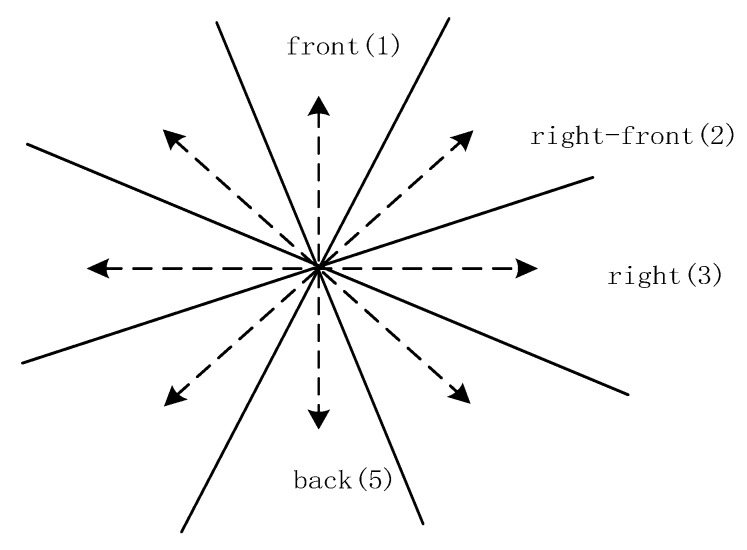
Illustration of path_(Θ)_. The dashed lines are the centerlines of corresponding cones. Each centerline is assigned a number from 1 to 8 clockwise (e.g., front is assigned 1).

As shown in Figure 8, path_(Θ)_ in Equation (2) is the minimum path between the centerlines of corresponding cones. For example, from front to left, path_(Θ)_ = 2. The visual field is divided into eight sectors, namely, “front, back, left, right, right front, right back, left front, and left back” or “north, south, west, east, northeast, northwest, southwest, and southeast”.

The visual field can also be divided into four sectors, namely, “front, back, left, and right” or “north, south, west, and east”. Then, the fuzzy relative direction membership function changes to Equation (3):

(3) μreldir(Θ)={1π4+|π2×path(Θ)−Θ|π4−a0 |π2×path(Θ)−Θ|≤aa≤|π2×path(Θ)−Θ|≤π4|π2×path(Θ)−Θ|≥π4

## 4. Positioning Localities Based on Probability Function

The process of obtaining the location region is introduced in this section. A joint probability function is proposed to describe the probability distribution of locality description in the region. Several definitions and a restriction are provided.

### 4.1. Location Region: Admissible Domain

**Figure 9:**
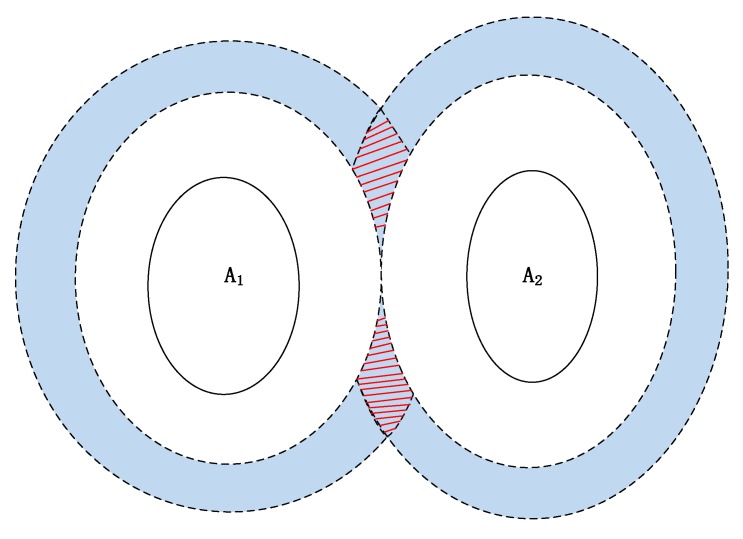
Definition of fuzzy band and admissible domain. The blue bands correspond to the fuzzy bands of objects A_1_ and A_2_ (e.g., Fuzzy_Band(A_1_), Fuzzy_Band(A_2_)); the red dashed regions correspond to the admissible domain (e.g., Admiss_Dom(A_1_,A_2_)).

**Definition** **1.** ***Fuzzy band:** Uncertain ring around the RO with fuzzy distance. The fuzzy band usually comprises outer and inner rings with upper and lower distances, as shown in Figure 9*.

**Definition** **2.** ***Admissible domain:** The fuzzy region in which the descriptive locality may be located; it is the intersection of the fuzzy bands of two or more ROs, as shown in Figure 9*.

By convention, most locality descriptions with distances and directions contain at most three ROs [9]. While describing locality with one RO, one relative direction relation is impossible to identify, so we don’t take this situation into consideration. Three fuzzy bands intersect in a unique admissible domain. However, the intersection of two fuzzy bands has two admissible domains, which is unacceptable for positioning localities. A unique region is necessary to satisfy the requirement of positioning localities.

**Figure 10:**
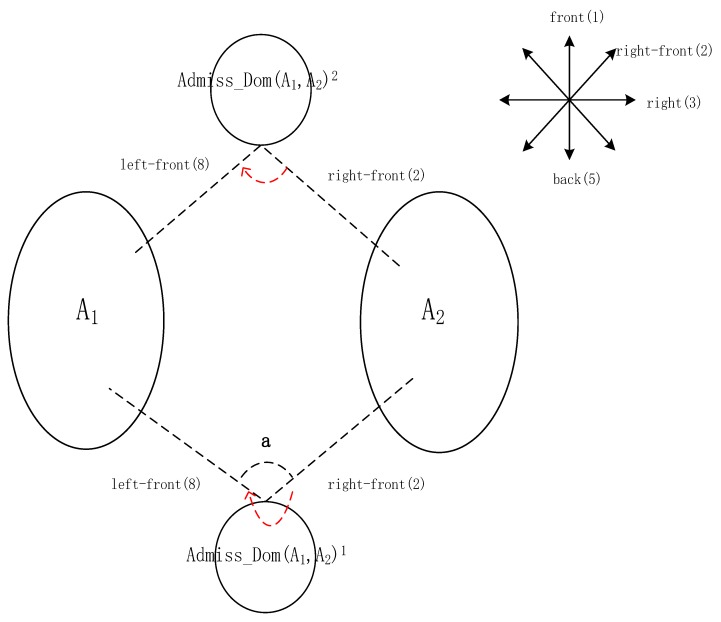
Illustration of the process of obtaining a unique admissible domain with two fuzzy bands: Admiss_Dom(A_1_,A_2_)^1^ (the direction of rotation is marked with a red dashed line).

The process of obtaining a unique admissible domain is detailed hereafter. We assume that the scene of locality description is as follows: “my front–right 50 m is A_2_, and my front–left 50 m is A_1_”. As shown in Figure 10, eight directions from front to left–front clockwise are assigned corresponding numbers from 1 to 8. The path(a) is the path between two direction lines. The admissible domains, intersected by the fuzzy bands of A_1_ and A_2_, are Admiss_Dom(A_1_,A_2_)^1^ and Admiss_Dom(A_1_,A_2_)^2^. Line 8 and 2 connect A_1_ and A_2_ to the admissible domains respectively. The unique admissible domain should meet the requirement that the direction from front–right (2) to front–left (8) is clockwise and path_(__a)_ = 6, that is, Admiss_Dom(A_1_,A_2_)^1^.

### 4.2. Probability Distribution: Joint Probability Function

**Figure 11:**
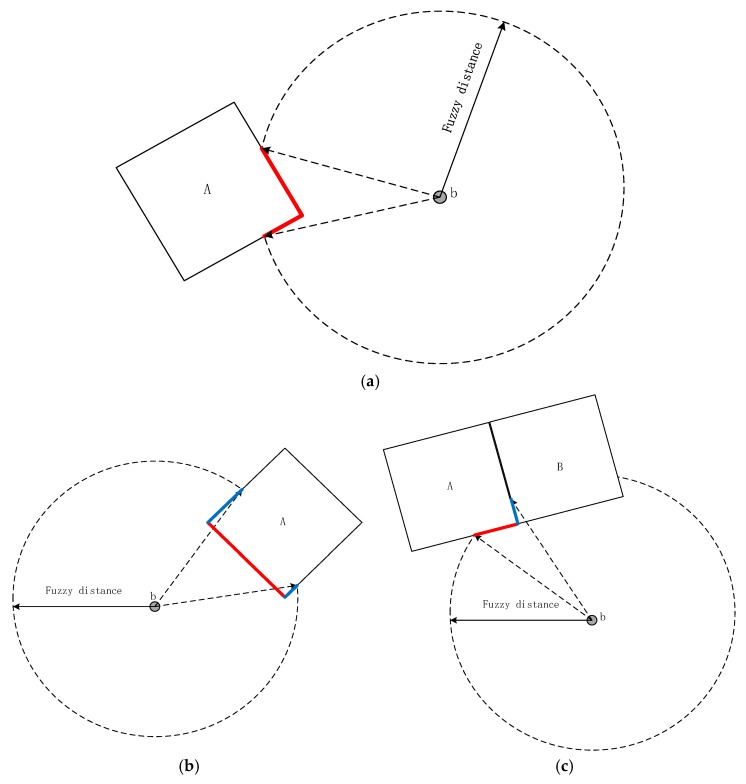
Definition of visible segment Visible_Seg(A) (red line). The red and blue lines form the boundary of A from a fuzzy distance locality b. The blue line is the invisible segment, and the red line is the visible segment. (**a**) The whole part; (**b**) due to invisibility itself; and (**c**) interrupted by RO B.

**Definition** **3.** **Visible segment:** The segment boundary of RO (Figure 11) is observed from a fuzzy distance, which is consistent with spatial cognition.

When viewed from a fuzzy distance, a segment of an RO should be in the visual field and possess the characteristic of visibility. From an algorithmic point of view, the visible segment reduces the number of points to be explored [13]. The points belong to the visible segment. The visible segment in Figure 11a contains the entire boundary of object A within a fuzzy distance. However, given the restriction of visibility, the boundary within a fuzzy distance does not completely belong to a visible segment in Figure 11b,c.

Certain restrictions (Figure 11) that are consistent with cognition should be proposed when exploring points in the visible segment.

**Figure 12:**
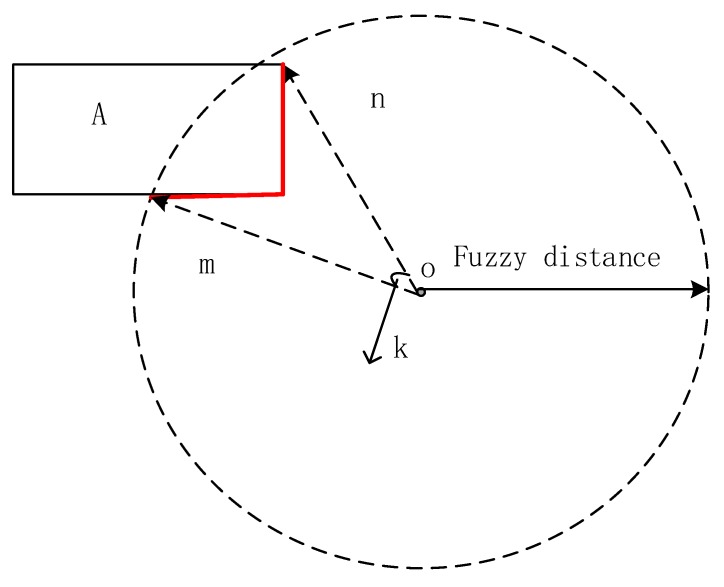
Restrictions of visible segment A; angle K should meet the restriction.

**Restriction:** The angle of sight should not exceed a concrete angle based on different cone-based models, in which the number of cones could be 4 or 8 [18]. Its value should be set based on fuzzy cognition and the Pareto principle that roughly 80% of effects originate from 20% of the cause. For example, in Figure 12, if the space is divided into eight cones, the angle of each cone is 45 degree. The occupation angle of the red line in its cone should be about 9 degree.

A locality description generally contains two or three ROs with associated spatial relations. Refinement can be performed to handle cases in which more than one spatial predicate and RO are involved [5]. We regard an object as a set of points, namely, A = {a_1_, a_2_, …, a_n_}. For a ∈ Visible_Seg(A), b ∈ Visible_Seg(B), and t ∈ T, we let dis(a,b) and dir(a,t,b) denote the distance and angle between two directions, respectively. A and B are ROs, and T represents the admissible domain. The Visible_Seg(A) and Visible_Seg(B) are the segment of A and B that meet the restriction.

We use positioning localities with two ROs as an example. We assume that the unique admissible domain (Admiss_Dom(A,B) = T) has been obtained. The calculation of the locality probability distribution in the admissible domain is as follows:

(1) We obtain Visible_Seg(A) and Visible_Seg(B) from locality t with upper fuzzy distance t ∈ Admiss_Dom(A,B).
(2) Refinement is performed to calculate distance probability P_dis_(t) with two ROs. P_dis_^A^(t) is the membership degree that maps the average dis(a,t) via the distance membership function Equation (1), that is, a ∈ Visible_Seg(A):
  (4) $$P_{\mathrm{dis}(t)}={P^{A}}_{\mathrm{dis}(t)}{P^{B}}_{\mathrm{dis}(t)}$$
(3) We calculate direction probability P_dir_(t). P^AB^_dir_(t) is the membership degree that maps the average dir(a,t,b) via the relative direction membership function Equation (2), that is, a ∈ Visible_Seg(A) and b ∈ Visible_Seg(B):
  (5) $$P_{\mathrm{dir}(t)}={P^{\mathrm{AB}}}_{\mathrm{dir}(t)}$$
(4) Refinement is performed to calculate joint probability P_(t)_:
  (6) $$P_{\left(t\right)}=P_{\mathrm{dir}(t)}P_{\mathrm{dis}(t)}$$

Having two ROs is only slightly different from having three ROs. The process is as follows:

(1) We obtain Visible_Seg(A), Visible_Seg(B), and Visible_Seg(C) from locality t with upper fuzzy distance t ∈ Admiss_Dom(A,B,C).
(2) We calculate distance probability P_dis_(t) with Equation (1):
  (7) $$P_{\mathrm{dis}(t)}={P^{A}}_{\mathrm{dis}(t)}{P^{B}}_{\mathrm{dis}(t)}{P^{C}}_{\mathrm{dis}(t)}$$
(3) We calculate direction probability P_dir_(t) with Equation (2):
  (8) $$P_{\mathrm{dir}(t)}={P^{\mathrm{AB}}}_{\mathrm{dir}(t)}{P^{\mathrm{BC}}}_{\mathrm{dir}(t)}{P^{\mathrm{AC}}}_{\mathrm{dir}(t)}$$
(4) Joint probability P_(t)_ is obtained with Equation (6):

This procedure introduces a calculation with more than one RO and spatial relation. For generality, we provide the PDF (Q_(t)_) from Equation (6) on the basis of [5]:

(9) $$Q_{\left(t\right)}=\frac{P_{\mathrm{dir}(t)}P_{\mathrm{dis}(t)}}{\sum_{i\in \mathrm{Admiss}\_\mathrm{Dom}}P_{\mathrm{dir}(i)}P_{\mathrm{dis}(i)}}$$

## 5. Case Study

To illustrate the process of positioning localities with distance and direction, we conducted two groups of cognitive experiments on the basis of the scenes presented in Section 4.2. The cognitive experiments are conducted in the same shopping market mentioned in Section 3.1. The shopping market has about 45 m visual space. Before the cognitive experiments, we select two points arbitrarily and mark them as TO(A) and TO(B). During the cognitive experiments, the participants, standing at the marked points, are asked to look around and describe their positions with distances and directions (i.e., front, left–front, and back). To ensure reasonable spatial cognition, we select male and female participants with different backgrounds, and their ages range from 20 to 60.

The first step of positioning is to find the admissible domain. On the basis of the distance cognition experiment, we adopt a 98% confidence interval as the upper and lower bounds of fuzzy distance [24], and their 98% confidence intervals (i.e., 10, 30 and 50 m) are (9.1, 12.2), (27.8, 38), and (49.5, 59.7), respectively. On the basis of the cognition experiment, the parameters (α, β, γ, δ) of 15 m and 20 m in Equation (1) are obtained by interpolation and they are as follows: 15 m (α = 5.5, β = 13, γ = 18, δ = 33.6) and 20 m (α = 7.4, β = 16, γ = 25, δ = 45.6). In these examples, the range of parameter a in Equation (2) is [2, 5] multiplied by path_(Θ)_.

An angle value should be determined to meet the restriction. Without additional contextual information, we cannot determine which cone-based model the relationship “front” stands for [5]. However, the relationship “left–front” represents the 8 cone-based model. Hence, for a direction relationship that lacks contextual information, we use the 4 cone-based model. The angle value that meets the restriction should be roughly 10° and 20° for the 8 and 4 cone-based models, respectively.

**Figure 13:**
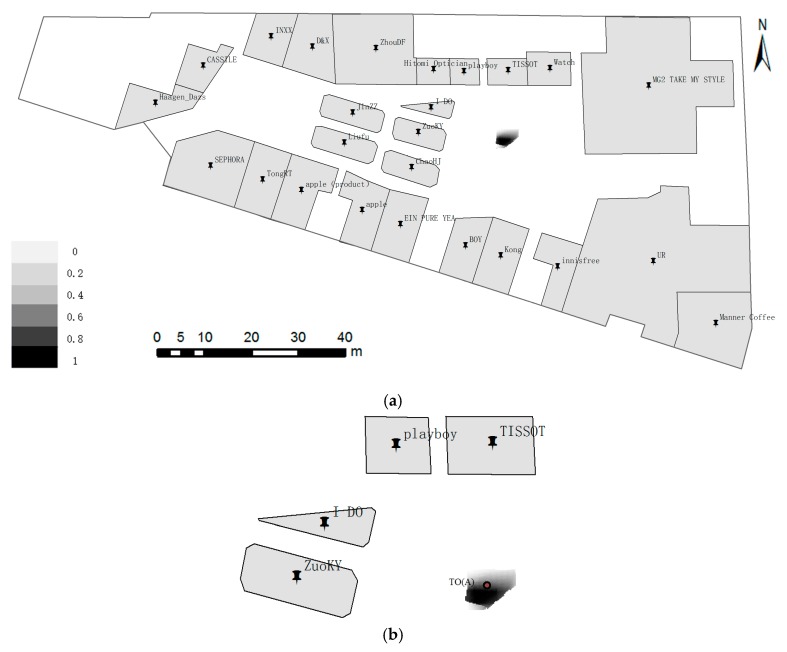
Positioning with two ROs at point TO(A) approximately 15.5 m away from TISSOT. The locality description is “front 20 m is TISSOT, and left 15 m is ZuoKY”. (**a**) Global; and (**b**) Local.

**Example** **1.** Positioning with two ROs standing at point TO(A). As shown in Figure 13, the locality description is “front 20 m is TISSOT, and left 15 m is ZuoKY”. Figure 13b shows a local map that depicts the locality probability distribution in the admissible domain (dashed region). The TO(A) in Figure 13b is inside the admissible domain, because the deviation of distances and directions in locality description are small. The lower-middle part of the admissible domain with a dark color reflects the most probable locality, and its position relative to TISSOT and ZuoKY is nearly at a 90° angle, which is consistent with spatial cognition.

**Figure 14:**
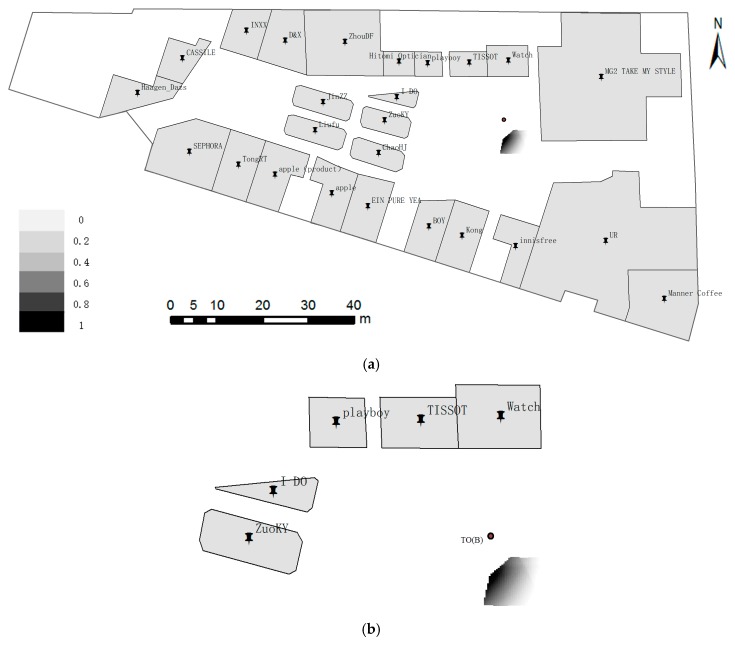
Positioning with three ROs at point TO(B) approximately 14.4 m away from Watch. The locality description is “front 20 m is Watch, left–front 30 m is Playboy, and left 30 m is ZuoKY”. (**a**) Global; and (**b**) Local.

**Example** **2.** Positioning with three ROs standing at point TO(B). As shown in Figure 14, the locality description is “front 20 m is Watch, left–front 30 m is Playboy, and left 30 m is ZuoKY”. Figure 14b indicates that the left part of the admissible domain, which has a dark color, has the most probable locality that meets the spatial relationship (i.e., distance and direction) from spatial cognition. The TO(B) in Figure 14b is out of the admissible domain, because the deviation of distances or directions in locality description are a bit large.

**Figure 15:**
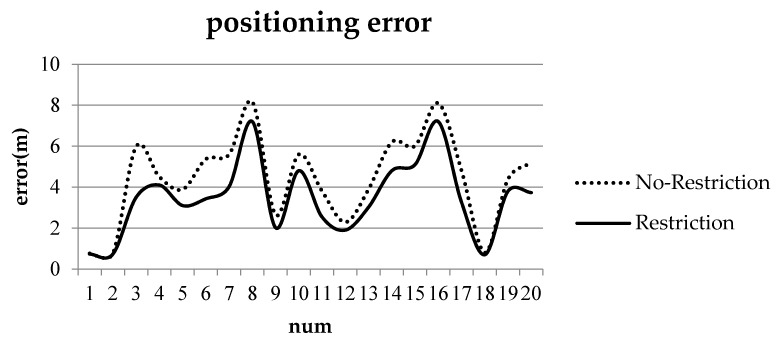
Positioning errors with two ROs: the dashed line indicates positioning errors with no restriction, and the solid line indicates positioning errors with restriction.

**Figure 16:**
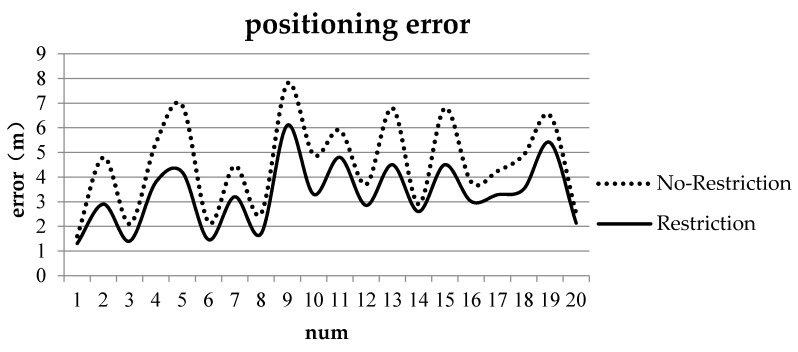
Positioning errors with three ROs: the dashed line indicates positioning errors with no restriction, and the solid line indicates positioning errors with restriction.

To verify the positioning accuracy of the model, we conduct two groups of cognitive experiments (positioning with two and three ROs). Table 1 and Table 2 show the locality descriptions at TO(A) and TO(B) with two and three ROs, respectively. Positioning error is expressed as the distance of the maximum probability point or center point of the maximum probability in the admissible region to the known point TO(A) or TO(B). The positioning errors are shown in Figure 15 and Figure 16.

As shown in Figure 15, the maximum and minimum positioning errors without restriction are 8.2 and 0.76 m, respectively, and the mean positioning error is 4.43 m. The maximum and minimum positioning errors with restrictions are 7.1 and 0.73 m, respectively, and the mean positioning error is 3.48 m.

As shown in Figure 16, the maximum and minimum positioning errors without restriction are 7.8 and 1.6 m, respectively, and the mean positioning error is 4.53 m. The maximum and minimum positioning errors with restrictions are 6.1 and 1.3 m, respectively, and the mean positioning error is 3.26 m. 

Figure 15 and Figure 16 indicate that the positioning errors are reduced by 0.95 and 1.27 m, respectively, when restrictions are considered. Angle restriction is not only consistent with cognition but can also improve the positioning accuracy. In practice, the restriction can be adjusted or ignored according to ROs and space extent.

Uncertainty is an inherent characteristic of spatial cognition [25]. Uncertainty in locality description originates from numerous sources, including external or internal factors, such as size, height, spatial distribution, task, and interest. As shown in Table 1 in numbers 6 and 8, different people possess different direction cognitions of the same scene. The distance descriptions in Table 1 (number 16) and Table 2 (number 9) are significantly large and cause considerable positioning errors. In a word, the more precise a locality description is with distances and directions, the more accurate the positioning is. Given the complex real environment and naïve cognition about distance and direction relations, the positioning accuracy exceeds 3.5 m, which is acceptable compared with that of complex and costly indoor positioning techniques [26] whose positioning accuracy is about 3–5 m when common smartphones are used.

Context (e.g., spatial and semantic) is an important factor in locality description [27]. Positioning accuracy would improve if numerous contexts, such as locality description that is likely to occur on roads or the presence of infrastructures in the admissible domain, are available.

## 6. Conclusions and Future Work

Positioning localities via locality description is a topic of next-generation GIS [8]. Resolution of positioning localities with place names and spatial relations facilitates the development of human-like geographic services that communicate with people intelligently about their everyday spatial needs [28]. 

To achieve positioning of localities indoors with cognitive distance and direction relationships semantically derived from locality description, we model relationships using the notions of admissible domain, visible segment, and restrictions. A joint probability function (i.e., distance and relative direction membership functions) is presented to describe the locality probability distribution in the admissible domain. The study demonstrates that the positioning accuracy exceeds 3.5 m within a 45 m visual space indoors. The contributions of this work are as follows:

(1) The intersection of the rings around ROs is modeled to the region (i.e., admissible domain) for location description. Two regions are commonly found in two ROs; the unique region can be selected.
(2) A cognitive experiment based on distance is conducted to obtain the width of rings, and a distance membership function is constructed to describe how far a locality is from the RO.
(3) To access the degree-to-direction relationship of a locality relative to ROs, we develop a novel relative direction membership function that is consistent with human spatial intuition.
(4) A joint probability function based on distance and relative direction membership functions is provided to determine the position degree. For consistency with intuition, we provide the notion of visible segment and its restrictions.

The model proposed in this work is based on spatial cognition and visibility. The membership function for semi-quantitative distance is based on statistics and does not consider contextual data, such as task, personal reputation, background, interest, and hobbies, because these personal data are difficult to obtain. If these data are available, a membership function based on ordered logit regression can be conducted. Then, positioning accuracy would improve significantly. The positioning accuracy in this work is for 45 m of visual indoor space. The larger the visual space is, the lower the positioning accuracy is because a large cognitive distance corresponds to a large deviation from the correct distance.

Similar to semi-quantitative distance, qualitative distance (i.e., “near”) is also frequently used in daily communication. Therefore, to make positioning localities with position description complete, a membership function based on qualitative distance will be used in our future work.

## Figures and Tables

**Table 1:** Locality description with two ROs.

TO	Num	RO1			RO2		
		Name	Distance	Direction	Name	Distance	Direction
A	1	ZuoKY	15	front	TISSOT	15	right
	2	ZuoKY	15	left	TISSOT	15	front
	3	ZuoKY	10	front	TISSOT	15	right
	4	I DO	20	front	ChaoHJ	25	left
	5	ZuoKY	20	left	TISSOT	20	front
	6	Playboy	20	right-front	ZuoKY	15	front
	7	I DO	15	front	ChaoHJ	20	left
	8	Playboy	25	front	ZuoKY	20	left
	9	I DO	15	left-front	TISSOT	15	front
	10	ZuoKY	15	front	TISSOT	20	right
B	11	Watch	15	front	Playboy	25	right-front
	12	Watch	10	front	Playboy	20	right-front
	13	Watch	15	right-front	Playboy	20	front
	14	Watch	20	front	Playboy	30	right-front
	15	Watch	20	front	ZuoKY	30	left
	16	Watch	25	front	ZuoKY	30	left
	17	Playboy	30	right-front	ZuoKY	30	front
	18	Playboy	25	right-front	ZuoKY	25	front
	19	ChaoHJ	30	right	I DO	20	front
	20	ChaoHJ	40	right-front	I DO	30	front

**Table 2:** Locality description with three ROs.

TO	Num	RO1			RO2			RO3		
		Name	Distance	Direction	Name	Distance	Direction	Name	Distance	Direction
A	1	ZuoKY	15	left	TISSOT	15	front	Hitomi Optician	20	left-front
	2	ZuoKY	10	left	TISSOT	15	front	Hitomi Optician	20	left-front
	3	ZuoKY	15	front	TISSOT	15	left	Hitomi Optician	25	left-front
	4	ZuoKY	20	left	TISSOT	25	front	Hitomi Optician	30	left-front
	5	ZuoKY	15	left	TISSOT	20	front	I DO	15	left-front
	6	ZuoKY	15	front	TISSOT	15	left	I DO	15	left-front
	7	ZuoKY	20	left-front	TISSOT	20	right-front	I DO	20	front
	8	ZuoKY	15	left-front	TISSOT	15	right-front	Playboy	20	front
	9	ZuoKY	20	left-front	TISSOT	20	right-front	Playboy	30	front
	10	ZuoKY	15	left-front	TISSOT	15	right-front	Playboy	25	front
B	11	I DO	30	front	ChaoHJ	30	left-front	TISSOT	20	right-front
	12	I DO	25	front	ChaoHJ	30	left-front	TISSOT	15	right-front
	13	Watch	20	front	ZuoKY	30	left	Playboy	30	left-front
	14	Watch	15	right	ZuoKY	25	front	Playboy	25	left-front
	15	Watch	20	right-front	ZuoKY	30	left-front	Playboy	30	front
	16	Watch	20	left	Playboy	30	right-front	I DO	30	front
	17	Watch	10	front	ZuoKY	25	left	Playboy	25	left-front
	18	Watch	15	right-front	ZuoKY	30	left-front	Playboy	30	front
	19	I DO	30	right	ChaoHJ	35	left	TISSOT	20	front
	20	Watch	15	left	ChaoHJ	25	right-front	I DO	25	front
